# Supplementary material for: The regulatory genome constrains protein sequence evolution: implications for the search for disease-associated genes
Source: PeerJ. 2020 Jul 21;8:e9554. doi: 10.7717/peerj.9554 (PMC7380284; doi:10.7717/peerj.9554)
Supplement: Supplemental Information 1 [file peerj-08-9554-s001.pdf]

SUPPLEMENTARY INFORMATION

The regulatory genome constrains protein sequence evolution: Implications for the search for disease-associated genes

Patrick Evans[1,*], Nancy J. Cox[1], Eric R. Gamazon[1,2,3,4 *]

[1]Division of Genetic Medicine, Vanderbilt University Medical Center, Nashville, TN 37232

[2]Data Science Institute, Vanderbilt University, Nashville, TN 37232

[3]Clare Hall, University of Cambridge, Cambridge, CB3 9AL, United Kingdom

[4]MRC Epidemiology Unit, University of Cambridge, Cambridge, CB2 0QQ, United Kingdom

Send correspondence to:

Patrick Evans, Ph.D. <patrick.evans@vanderbilt.edu>

Eric R. Gamazon, Ph.D. <ericgamazon@gmail.com> (Lead Contact)

Running title: Evolutionary rate and the search for disease genes

Keywords: evolution, transcriptome, heritability, gene expression, imputation, PrediXcan, TWAS, evolutionary rate, conserved genes, Mendelian disease genes

Local Genetic Control and Evolutionary Rate

Heritability provides a measure of the potential of a trait to respond to selection (Roff, 2000). Gene expression level as a quantitative trait has a heritable component and for many genes, expression level can be predicted, to a degree measured by heritability and in a tissue-dependent manner, based on genetic polymorphism data (Gamazon et al., 2015). We, therefore, set out to quantify the contribution of the degree of genetic control of gene expression to evolutionary rate. In particular, this analysis would show the extent to which the degree of genetic control correlates with evidence for purifying selection (dN/dS<1) or positive selection (dN/dS>1) (or relaxed constraint). Because current sample sizes still have insufficient power to detect *trans* expression quantitative trait loci (eQTLs) in available transcriptome data, we focus primarily on local (*cis*) regulation of gene expression (using the residual after adjusting for hidden confounders; see Methods). For heritability estimated in the DGN data set (whole blood, N=922) (Battle et al., 2014), we find a significant correlation with dN/dS (Spearman's $\rho=0.092$, $p=4.4\times10^{-21}$), dN (Spearman's $\rho=0.154$, $p=5.1\times10^{-60}$), and dS (Spearman's $\rho=0.136$, $p=1.8\times10^{-47}$), using the human-chimp comparison. (These estimates are conservative relative to the human-mouse comparison.) Thus, conserved genes tend to have lower *cis* heritability than other genes (Figure 2C for comparison with fast-evolving genes in whole blood transcriptome, Mann-Whitney U $p=1.2\times10^{-17}$). Since heritability is the ratio of genetic variance to phenotypic variance, conserved genes may show lower *cis* heritability due to lower genetic variance from local variation, larger phenotypic variance, or both. For example, for conserved genes in GTEx skeletal muscle tissue, *cis* eQTL effect sizes are significantly lower (Spearman's $\rho=0.204$ between effect size and dN/dS, $p=1.3\ 10^{-133}$) while expression variance is significantly larger (Spearman's $\rho=-0.270$, $p=3.7\times10^{-267}$).

EPRCA shows that the correlation between dN/dS and heritability (estimated in DGN whole blood) after controlling for MaxVariance remains significant (Spearman's $\rho=0.097$, permutation

p<0.001). Similarly, the correlation between dN/dS and MaxVariance after adjusting for heritability is significant (Spearman's $\rho=-0.119$, permutation $p<0.001$). These results provide strong evidence for independent contributions to variance in evolutionary rate from these features. Furthermore, we find a modest but significant correlation between heritability and MaxVariance (Spearman's $\rho=0.038$, $p=4.04\times10^{-5}$), but not maximum expression level.

Branch assignment

Gene age estimation is a complex problem with different domains of a gene possibly having different ages (Capra et al., 2013) and, in addition, requires caution in the interpretation of trends given the possibility of phylostratigraphic bias (Moyers & Zhang, 2017). We utilized gene branch assignments based on orthologous presence in the vertebrate phylogeny, as previously described (Zhang et al., 2010). This approach tended to identify young genes more conservatively than a previous approach (Church et al., 2009). The final gene branch assignments included 17,162 protein-coding genes, 182 lincRNAs, and 806 pseudogenes (GENCODE (Harrow et al., 2012) annotation v19). Unless otherwise stated, we restricted the analysis to protein-coding genes. Of the protein-coding genes, 700 were primate-specific, mapping to the Rhesus-Orangutan-Chimp-Human phylogenetic branches (i.e., between branches 8 and 12; Supplementary Table 3). Branches are ordered from 0 to 12, with branch 0 being the oldest and indicative of a gene shared among vertebrates, while branch 12 is human-specific.

We compared branch assignment to expression for each tissue (Supplementary Figure 3). Highly expressed genes tend to be evolutionarily old (i.e., map to lower branch number), while newer genes tend to have lower expression (Spearman's $\rho=-0.47$ to -0.31, $p<2.2\times10^{-16}$). Brain tissues tend to show the strongest effect (Spearman's $\rho=-0.47$ to -0.45), in terms of variance explained, of branch assignment on expression level, with the cortical tissues having the highest absolute magnitude. Testis shows the lowest absolute effect (Spearman's $\rho=-0.31$), with branch assignment explaining two thirds of the

variability in expression level relative to brain. Whole blood is the next lowest (Spearman's ρ=-0.357), once again reinforcing the observation that the transcriptome in the most accessible tissue is an outlier in key aspects of protein sequence evolution. Although the effect of branch assignment on expression level and on expression variance across tissues is highly correlated (Spearman's ρ=0.975, $p<2.2 \times 10^{-16}$), gene age accounts for a significantly greater proportion of the variability in expression level than in expression variance (median of 19.5% vs 16.5%, Mann-Whitney $p=3.7 \times 10^{-7}$).

Branch assignment significantly determines expression breadth (Spearman's ρ=0.24 between branch and τ, $p<2.2 \times 10^{-16}$), which indicates increasing broadness of expression of a gene throughout its evolutionary lifespan.

Gene network node properties

The extent of integration into gene networks may constrain protein sequence evolution. Perturbation of genes central to a biophysical network may imply a greater fitness cost, as such perturbation can lead to downstream effects across multiple gene networks. Here we utilized the STRING protein-protein interaction network (see Methods). The 100 genes with the highest degree (each with at least 1000 interaction partners) are enriched for adenosine 5'-triphosphate (ATP) binding (n=48, Benjamini-Hochberg adjusted $p=6.5 \times 10^{-27}$) and actin-related proteins (n=21, Benjamini-Hochberg adjusted $p=1.0 \times 10^{-32}$) crucial to the formation of the cytoskeleton.

Widely expressed genes tend to have more interactions with other genes (Spearman's ρ=-0.1855 between τ and node degree, $p<2.2 \times 10^{-16}$). The number of interactions significantly constrains sequence evolution (Spearman's ρ=-0.06 between dN/dS and node degree, $p=4.667 \times 10^{-10}$). We did find a unique gene, Uridine Monophosphate Synthetase (UMPS), with more than 1,300 interaction partners and evidence for positive selection based on human-chimp divergence but not based on human-mouse divergence. Furthermore, the gene is of ancient origin (shared among vertebrates; branch 0) and

associated with a Mendelian disorder (OMIM 613891). This example not only illustrates the dependence of the evidence for positive selection on evolutionary time-scale, but also raises the hypothesis that adaptive evolution may work through biophysical networks, with genes under positive selection potentially interacting and genes in central network positions having a strong influence on fitness.

The variance in node degree observed for older genes (branches 0-3) is much larger than for younger genes (branches 4-12) ($F=1.247$, $p=8.242\times10^{-07}$), so that the genes with the largest number of interactions come from the oldest phylogenetic branches (Supplementary Figure 4). Genes on the oldest branch with few (1 to 10) interactions are enriched for homeodomain genes (Benjamini-Hochberg adjusted $p=5.61\times10^{-18}$).

We tested whether node degree and expression level independently constrain evolutionary rate. Gene connectivity and expression show no significant correlation ($p=0.33$) with each other. We find support for independent contribution from these features in our data (Spearman's $\rho=-0.1583$, permutation $p<0.001$, mean permutation null=-4.481×10^{-5}, std dev permutation null=0.011 for correlation of protein evolutionary rate with maximum expression level (across the tissues) while controlling for node degree and Spearman's $\rho=-0.0591$, permutation $p<0.001$, mean permutation null=-7.634×10^{-5}, std dev permutation null=0.012 for correlation with node degree while adjusting for maximum expression level).

Although network characteristics and topology clearly correlate significantly with expression variance in all tissues (maximum p-value=3.6×10^{-11} from the correlation with node degree), we observed a tissue dependence in the strength of the correlation between number of interaction partners and expression variance. Lung shows the lowest correlation (Spearman's $\rho=0.059$) despite having a relatively large sample size (n=238, Supplementary Table 1) while transformed lymphocytes (LCLs) (n=219) and cultured primary fibroblasts (n=85) show the highest correlations (Spearman's $\rho=0.21$,

p=2.07x10^{-125} and Spearman's ρ=0.19, p=9.6x10^{-98} respectively). Interestingly, the correlation for the primary tissues of origin for the LCLs (i.e., whole blood) and for the fibroblasts (i.e., fresh skin) show substantially lower correlation (Spearman's ρ=0.12, p<2.2x10^{-16} and Spearman's ρ=0.085, p<2.2x10^{-16}, respectively), suggesting global changes in expression profile, with important consequences for network constraints, in the cell lines.

Gene regulation and evolutionary rate on specific gene sets

We examined expression features and protein evolutionary rate in specific sets of genes compared to their complement in the set of protein-coding genes (Supplementary Table 5). Mendelian disease genes show higher expression level (Mann-Whitney W=12961000, p<2.2x10^{-16}) and lower protein evolutionary rate (W=13293000, p=7.68x10^{-13}) than other protein-coding genes. However, unexpectedly, Mendelian disease genes do not show greater expression breadth or node degree in PPI networks. Conversely, LOF-tolerant genes show higher protein evolutionary rate (W=439220, p=4.92x10^{-06}) and greater tissue specificity of expression (W=696830, p=8x10^{-4}) than other protein-coding genes. Essential genes are of significantly more ancient origin (W=1969600, p=1.924x10^{-8}) and under greater purifying selection (W=1590800, p=1.236x10^{-12}) than Mendelian disease genes. Furthermore, essential genes show higher expression level (W=25579000, p<2.2x10^{-16}), greater expression breadth (W=17996000, p<2.2x10^{-16}), and higher connectivity (W= 10663000, p=0.01) than other protein-coding genes. We find (using random sampling [N=1000] without replacement of protein-coding genes of the same count) that Mendelian disease genes are enriched in the oldest branches (0 through 3) and show a depletion in the subsequent branches of the phylogeny (4 through 12) (Supplementary Figure 5). The branch in which the transition from enrichment to depletion for Mendelian disease genes is observed (branch 3) separates mammals from other vertebrates, suggesting that Mendelian disease genes may largely predate the emergence of mammals. However, given the spurious findings that may arise from

the method of gene age estimation, analysis restricted to “error-resistant genes” should reduce phylostratigraphic bias (Moyers & Zhang, 2017).

Finally, we analyzed genes that interact with the environment: immune response genes and olfactory genes. Immune response genes show significantly higher expression ($W=9641000$, $p<2.2x10^{-16}$) and expression breadth ($W=11596000$, $p=1.34x10^{-09}$), but fewer network interactions ($W=5991100$, $p=0.0002$) than other protein-coding genes, but do not show significantly higher evolutionary rate. Adaptive immune response genes, however, do show significantly higher evolutionary rate ($W= 2115600$, $p=0.0006$). Olfactory genes have significantly lower expression ($W=7231900$, $p<2.2x10^{-16}$) and higher evolutionary rate ($W=971270$, $p<2.2x10^{-16}$) than the rest of the protein-coding genes, but also show a narrower expression breadth ($W=2165500$, $p<2.2x10^{-16}$).

We sought to utilize the spectrum of clinical manifestations represented by these sets of genes to probe potential mechanisms underlying the observed relationship between expression features and evolutionary rate. We found that Mendelian disease genes (Spearman’s $\rho=-0.078$, $p=0.002$) and essential genes (Spearman’s $\rho=-0.112$, $p=1.19x10^{-7}$) show substantially lower effect, in terms of variability explained, on evolutionary rate than the full set of proteins (Spearman’s ρ ranges from -0.34 to -0.17, $p<3.63x10^{-102}$). Using 1000 random sample sets of genes (of the same size as the list of Mendelian disease genes and the list of essential genes, separately analyzed, and matched on variation in expression level and variation in dN for these gene sets since these sets generally have lower such variations) drawn from the complete set of protein-coding genes, the observed correlation estimates are significantly higher than expected; no random set matches or exceeds the observed correlation estimate for each gene set (empirical $p<0.001$; Figure 4C). Thus, expression level explains a significantly lower proportion of the variance in evolutionary rate for Mendelian disease and essential genes. This result suggests that gene function may interact with expression level in influencing evolutionary rate

and that the important correlation may vary for gene sets across the genome depending on gene function. In support of this, we find that an interaction model that incorporates gene expression and LOF-tolerance provides a significantly better fit to the data than a regular regression model (anova Chi-square test $p=5.24 \times 10^{-5}$).

References

Battle A, Mostafavi S, Zhu X, Potash JB, Weissman MM, McCormick C, Haudenschild CD, Beckman KB, Shi J, Mei R, Urban AE, Montgomery SB, Levinson DF, Koller D. 2014. Characterizing the genetic basis of transcriptome diversity through RNA-sequencing of 922 individuals. *Genome Research* 24:14–24. DOI: 10.1101/gr.155192.113.

Capra JA, Stolzer M, Durand D, Pollard KS. 2013. How old is my gene? *Trends in genetics: TIG* 29:659–668. DOI: 10.1016/j.tig.2013.07.001.

Church DM, Goodstadt L, Hillier LW, Zody MC, Goldstein S, She X, Bult CJ, Agarwala R, Cherry JL, DiCuccio M, Hlavina W, Kapustin Y, Meric P, Maglott D, Birtle Z, Marques AC, Graves T, Zhou S, Teague B, Potamousis K, Churas C, Place M, Herschleb J, Runnheim R, Forrest D, Amos-Landgraf J, Schwartz DC, Cheng Z, Lindblad-Toh K, Eichler EE, Ponting CP, Mouse Genome Sequencing Consortium. 2009. Lineage-specific biology revealed by a finished genome assembly of the mouse. *PLoS biology* 7:e1000112. DOI: 10.1371/journal.pbio.1000112.

Gamazon ER, Wheeler HE, Shah KP, Mozaffari SV, Aquino-Michaels K, Carroll RJ, Eyler AE, Denny JC, GTEx Consortium, Nicolae DL, Cox NJ, Im HK. 2015. A gene-based association method for mapping traits using reference transcriptome data. *Nature Genetics* 47:1091–1098. DOI: 10.1038/ng.3367.

Harrow J, Frankish A, Gonzalez JM, Tapanari E, Diekhans M, Kokocinski F, Aken BL, Barrell D, Zadissa A, Searle S, Barnes I, Bignell A, Boychenko V, Hunt T, Kay M, Mukherjee G, Rajan J, Despacio-Reyes G, Saunders G, Steward C, Harte R, Lin M, Howald C, Tanzer A, Derrien T, Chrast J, Walters N, Balasubramanian S, Pei B, Tress M, Rodriguez JM, Ezkurdia I, van Baren J, Brent M, Haussler D, Kellis M, Valencia A, Reymond A, Gerstein M, Guigó R, Hubbard TJ. 2012. GENCODE: the reference human genome annotation for The ENCODE Project. *Genome Research* 22:1760–1774. DOI: 10.1101/gr.135350.111.

Moyers BA, Zhang J. 2017. Further Simulations and Analyses Demonstrate Open Problems of Phylostratigraphy. *Genome Biology and Evolution* 9:1519–1527. DOI: 10.1093/gbe/evx109.

Roff D. 2000. The evolution of the G matrix: selection or drift? *Heredity* 84 (Pt 2):135–142.

Zhang YE, Vibranovski MD, Landback P, Marais GAB, Long M. 2010. Chromosomal redistribution of male-biased genes in mammalian evolution with two bursts of gene gain on the X chromosome. *PLoS biology* 8. DOI: 10.1371/journal.pbio.1000494.

Supplementary Legends

Supplementary Figure 1. Expression breadth statistic τ by gene type for GENCODE v19 annotation.

Supplementary Figure 2. Density plot of τ in protein-coding (red) and lincRNA (blue).

Supplementary Figure 3. Comparison of gene age estimate and expression level and variance.

Supplementary Figure 4. Genes showing the largest number of interactions come from the oldest phylogenetic branches.

Supplementary Figure 5. Mendelian disease genes are enriched in the oldest branches (0 through 3) and show a depletion in the subsequent branches of the phylogeny (4 through 12).

Supplementary Table 1. Table of the number of samples in each tissue and genes that have dN and dS information for each tissue.

Supplementary Table 2. Summary statistics of correlation between expression level and protein evolutionary rates.

Supplementary Table 3. Input Data.

Supplementary Table 4. Trait model AIC values from univariate models based on human-mouse comparisons.

Supplementary Table 5. Mann-Whitney results of gene group comparisons.

Supplementary Table 6. Correlation between out-of-sample prediction R^2 for gene expression and evolutionary rate.
