# Supplementary figures and images for: The regulatory genome constrains protein sequence evolution: implications for the search for disease-associated genes
Source: PeerJ. 2020 Jul 21;8:e9554. doi: 10.7717/peerj.9554 (PMC7380284; doi:10.7717/peerj.9554)

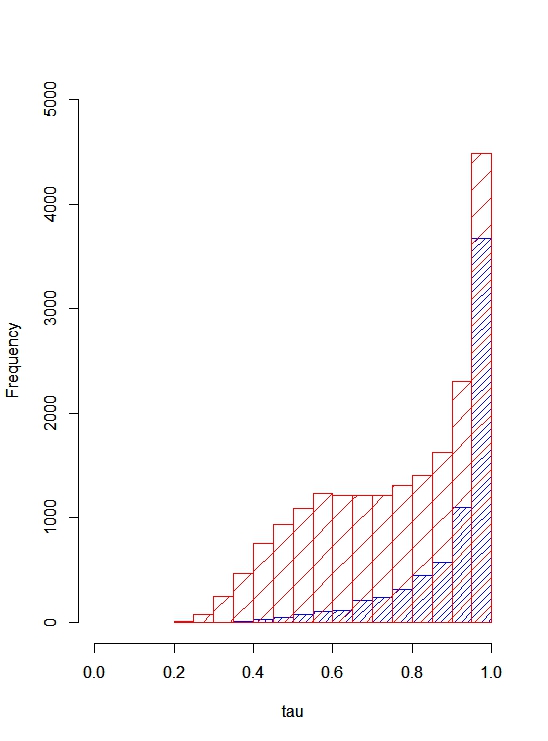

Supplement: Supplemental Information 9 [file peerj-08-9554-s009.jpeg]

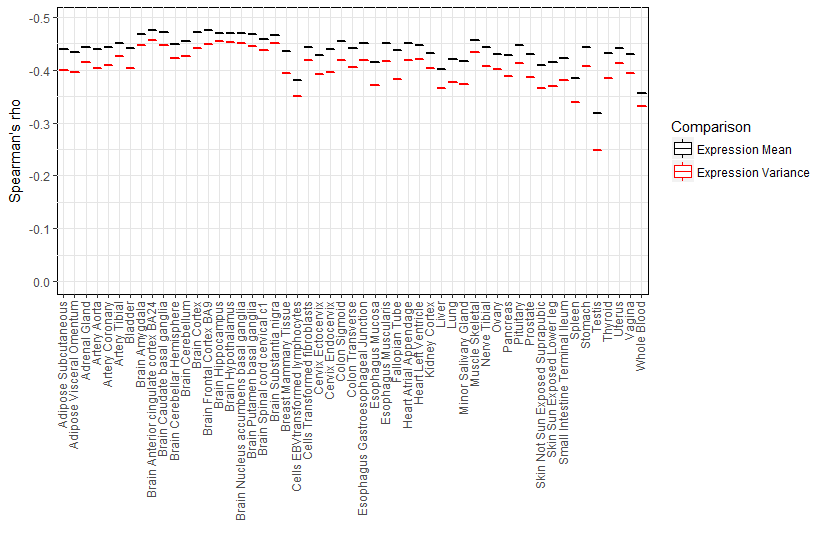

Supplement: Supplemental Information 10 [file peerj-08-9554-s010.png]

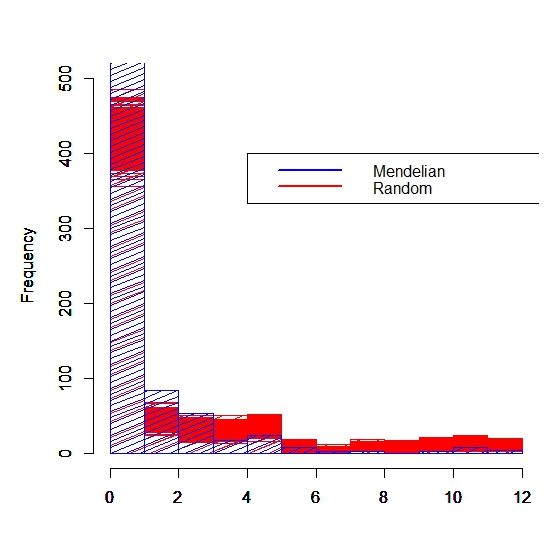

Supplement: Supplemental Information 12 [file peerj-08-9554-s012.jpeg]
